# [Retracted] Effect of enhanced expression of COL8A1 on lymphatic metastasis of hepatocellular carcinoma in mice

**DOI:** 10.3892/etm.2026.13098

**Published:** 2026-02-09

**Authors:** Zhen-Hai Ma, Jin-Hui Ma, Li Jia, Yong-Fu Zhao

Exp Ther Med 4:621–626, 2012; DOI: 10.3892/etm.2012.652

Following the publication of this paper, it was drawn to the Editor’s attention by a concerned reader that the control western blots shown in Fig. 1B had already appeared in a couple of other papers published by the same authors in *The International Journal of Biochemistry & Cell Biology* (which have subsequently been retracted), and also a third paper by the same research group submitted at around the same time as this article to the journal *IUBMB Life*. In addition, data shown in the RT-qPCR electrophoresis experiment in Fig. 1A (both the control data, and the RNA data of interest) had already appeared in another paper published by the same research group in 2007 in *The International Journal of Biochemistry & Cell Biology.*

Owing to the fact that the contentious data in the above article had already been published prior to the submission of this article to *Experimental and Therapeutic Medicine*, the Editor has decided that this paper should be retracted from the Journal. The authors were asked for an explanation to account for these concerns, but the Editorial Office did not receive a reply. The Editor apologizes to the readership for any inconvenience caused.

